# Extraluminal Colonic Carcinoma Invading into Kidney: A Case Report and Review of the Literature

**DOI:** 10.5402/2011/707154

**Published:** 2011-04-26

**Authors:** J. Nelson, K. Rinard, A. Haynes, S. Filleur, T. Nelius

**Affiliations:** ^1^Department of Urology, Texas Tech University Health Sciences Center, Lubbock, TX 79430-7260, USA; ^2^Department of Microbiology and Immunology, Texas Tech University Health Sciences Center, Lubbock, TX 79430, USA

## Abstract

Renal metastasis from primary colon cancer is very rare, comprising less than 3% of secondary renal neoplasms. There are just 11 cases reported in the medical literature of colonic adenocarcinoma metastatic to the kidney. Of these cases, none occurred via direct invasion. We report a unique case of a 51-year-old female with extraluminal colonic adenocarcinoma which directly invaded into the kidney. Additionally, we investigate the causal relationship between the site of invasion and a previous stab injury by reviewing the role of the peritoneum and Gerota's fascia in preventing the spread of metastatic cancer into the perirenal space. Due to the rarity of this event, we present this case including a review of the existing literature relative to the diagnosis and treatment.

## 1. Introduction

Colon cancer affects more than 150,000 Americans each year, and is responsible for approximately 10% of cancer mortality in the United States [1]. More than 90% of colon cancers arise sporadically via the adenoma-to-carcinoma sequence [2]. Hereditary polyposis and nonhereditary polyposis syndromes represent less than 10% of colon cancers [2]. The liver is the most common site of colon cancer metastases because the venous drainage from the colon enters the portal system. Once metastasis to the liver has occurred, the lungs, peritoneum, pelvis, and adrenals may also become involved [1]. Renal metastasis from primary colon cancer is very rare, comprising less than 3% of secondary renal neoplasms, and usually occurs via hematogenous spread [3]. A comprehensive MEDLINE search resulted in only 11 case reports within the medical literature of colonic adenocarcinoma metastatic to the kidney [3–13]. Of these cases, none occurred via direct invasion. We report a unique case of a 51-year-old female with extraluminal colonic adenocarcinoma that directly invaded into the kidney. Furthermore, we investigate the causal relationship between the site of invasion and a previous stab injury by reviewing the role of the peritoneum and Gerota's fascia in preventing the spread of metastatic cancer into the perirenal space.

## 2. Case Report

### 2.1. Initial Presentation

In January 2010, a 51-year-old female presented to a rural hospital with a one month history of left lower and upper abdominal quadrant pain. The patient was admitted to the hospital with the preliminary diagnosis of pancreatitis. After admission, the patient developed gross hematuria. It was discovered that the patient had a past medical history significant for a stab injury in 1998 to her left kidney with involvement of the left renal artery. At that time, the patient underwent a transabdominal/transperitoneal partial nephrectomy. Due to the patient's complicated past medical history and her present gross hematuria, the decision was made to transfer the patient to our service for a higher level of care.

### 2.2. Hospital Course

After admission to our hospital, a transurethral catheter was placed, which showed gross hematuria. Upon further evaluation, the decision was made to perform a cystoscopy and evacuation of blood clots. The urethrocystoscopy showed no gross pathology in the urethra and bladder. The left and right orifice were normal in size, place, and configuration. In order to locate the source of bleeding, bilateral ureteroscopy was performed. The right ureter and renal pelvis were found to be unremarkable. The left ureter also appeared to be free of pathology. However, at approximately the level of the ureteropelvic junction, a papillary mass was noted that appeared to extend into the left renal pelvis. A biopsy was unable to be obtained due to significant bleeding. After the blood clots were evacuated, and taking into account the past history of hematuria with a small, atretic kidney, it was deemed necessary to pass a stent into the left ureteral pelvis. The patient tolerated the procedure well. The results of the cystoscopy were discussed with the patient, and a nephrectomy was being considered to remove the tumor. However, after cystoscopy the patient developed an ileus. She was also very malnourished. Once her bleeding and ileus resolved, the decision was made to allow her to be discharged home to recuperate in order to better tolerate the nephrectomy. The patient was for a followup appointment two weeks later to evaluate for a nephrectomy. The patient did not show up for her appointment and was lost to followup.

Seven months later, the patient was admitted to the hospital complaining of progressive left flank and left-sided abdominal pain that gradually increased in intensity for four days. It was accompanied by nausea and vomiting, dysuria, and frequency. The patient stated that she had no bowel movement for 3 weeks. She also complained of poor appetite and weight loss, but did not know the amount of the weight loss over the past few months. 

On physical exam, the patient was found to be in moderate distress. She was alert and oriented with mild cachexia. The abdomen was soft, with moderate tenderness to palpation of the left side with the pain radiating to the left groin. The abdominal exam was negative for rebound tenderness and positive for bowel sounds. A surgical scar 3 inches in diameter was visible in midline. Two ventral hernias were also visible on the abdomen. The physical exam also showed costovertebral angle tenderness bilaterally, with marked tenderness on the left side. The patient also was found to be septic at that time. 

**Figure 1:**
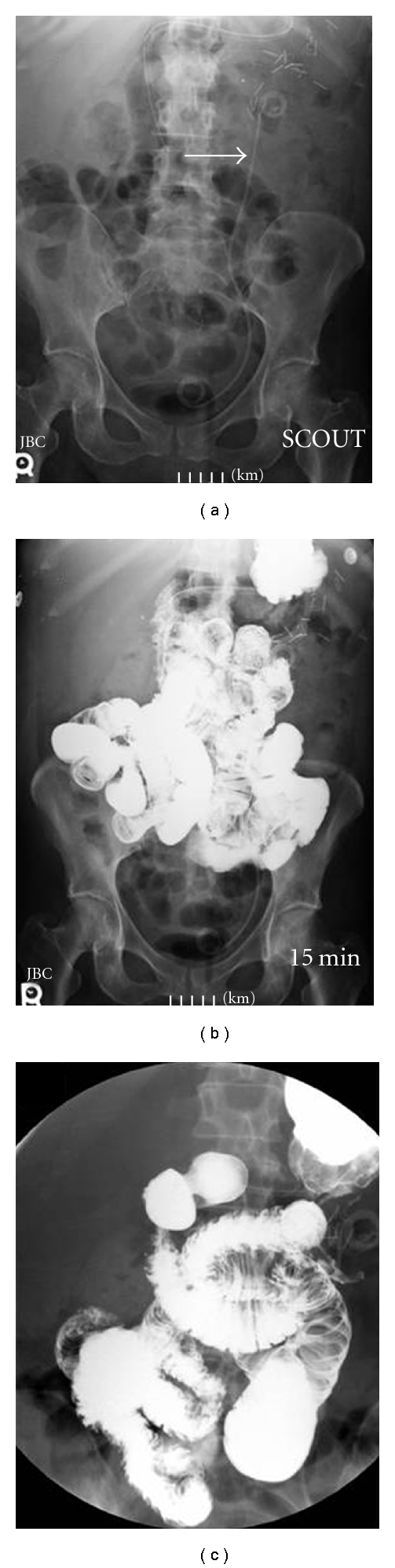
Gastrointestinal series with small bowel imaging. (a) Scout film showing Double-J Stent orthotopic in the left-sided renal collecting system (arrow). (b) Barium contrast media was injected through nasogastric tube under fluoroscopic evaluation. The proximal small bowel demonstrates intermittent mild dilatation. Obstructive point was not clearly identified. (c) Higher magnification.

**Figure 2:**
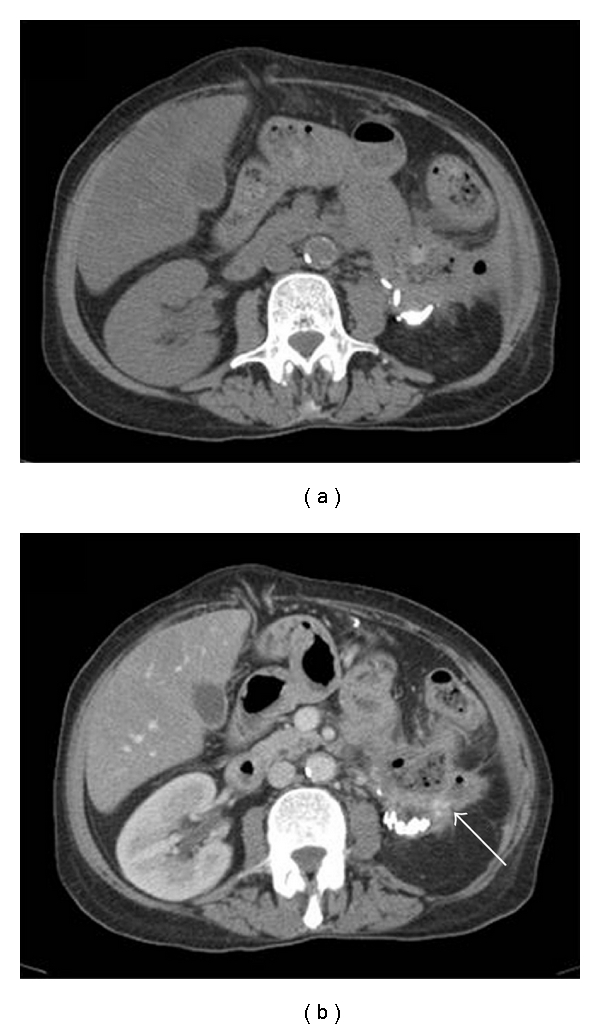
CT scan of the abdomen and pelvis. (a) Imaging without i.v. contrast. Stranding and thickening of the soft tissue immediately below the lower pole of the left kidney and around the splenic flexure. (b) Imaging after i.v. contrast application. Stranding and thickening of the soft tissue around the kidney appears more prominent (arrow).

For further evaluation of her newly diagnosed ileus, the patient underwent a gastrointestinal series with small bowel imaging. The proximal small bowel demonstrated intermittent mild dilatation. The obstructive point was not clearly identified. Contrast media extended through the jejunum and ileum without evidence of obstruction. The terminal ileum was normal in appearance (Figure 1). Additionally, a colonoscopy was performed which revealed a stricture at the site of the splenic flexure. No intraluminal tumor was encountered. A standard computed tomography (CT) scan of the abdomen and pelvis with and without IV contrast was obtained. The CT scans showed some stranding and thickening of the soft tissues immediately below the lower pole of the left kidney. There was also some stranding immediately around the splenic flexure that began immediately below the tail of the pancreas. No soft tissue density was present to suggest a mass. There was no evidence of bowel obstruction, but the small bowel in the abdomen was mildly distended and filled with fluid possibly representing mild ileus. The nephroureteral stent was noted on the left side in stable position (Figure 2). 

**Figure 3:**
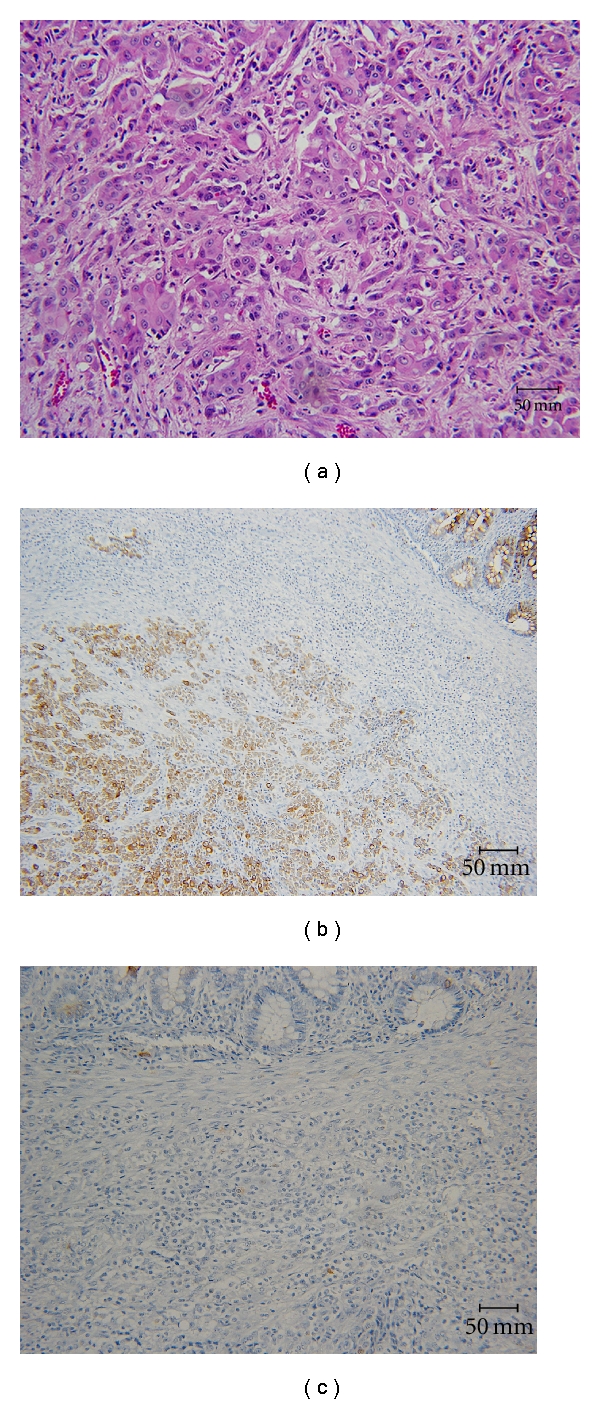
Histopathological examination. (a) Pathology slide demonstrating the poorly differentiated carcinoma of the colon (Hematoxylin and Eosin, 20x). Immunohistochemical stains for CK20 and CK7 were found to be uniformly CK20 positive (b) and CK7 negative (c).

At this point, the working diagnosis of a malignant tumor of the left-sided renal pelvis invading into the perirenal space with involvement of the descending colon and tail of the pancreas was made. Once the patient was stabilized, the left-sided ureteral stent was removed and the patient was optimized for an exploratory laparotomy. The patient underwent radical (en bloc) resection of the left kidney including partial colectomy, splenectomy, partial pancreatectomy, and partial small bowel resection. Intra-operatively, a large mass in her left upper quadrant was encountered that appeared to originate from the left kidney. The tumor was locally invasive, involving the very proximal jejunum, the splenic flexure of the colon, the left adrenal gland, and the distal aspect of the pancreas. This situation necessitated an en block resection. On gross pathology, the measurement of tumor size was not definite, but appeared to be 8 cm in maximal dimension. The specimen was partially necrotic and presumably obliterating the colon. The tumor did not involve the lumen of the colon or small intestine. Microscopic examination showed a poorly differentiated carcinoma of the colon invading into the kidney. Noteworthy, the tumor was only identifiable in the submucosal layer of the colon. Immunohistochemical stains for CK20 and CK7 were performed and found to be uniformly CK20 positive and CK7 negative (Figure 3). These findings strongly evince a colonic origin for the entire tumor. At this point, the diagnosis of an extraluminal colonic carcinoma invading into the kidney was made. Tumor stage according to the TNM-system was pT4b, pN1b, pM1a. After adequate recovery from surgery, the patient will undergo adjuvant chemotherapy.

## 3. Discussion

To the best of our knowledge, there is no documented or similar case published in the literature, where extraluminal colonic adenocarcinoma directly invaded into the kidney. Of the 11 case reports within the medical literature of colonic adenocarcinoma metastatic to the kidney, none occurred via direct invasion [3–13]. Another unique aspect of this case is the relationship between the site of tumor invasion and the patient's previous stab injury. The rarity of this event and its relationship to the site of a previous stab injury led us to investigate the role of the peritoneum and Gerota's fascia in preventing the spread of metastatic cancer into the perirenal space. 

The retroperitoneum is formed by three tissue layers: the inner, intermediate, and outer layers. The inner layer forms the visceral fascia, the intermediate layer forms the renal fascia, and the outer layer forms the fascia lining the body wall [14, 15]. The retroperitoneal space is the area of the posterior abdominal wall that is located between the parietal peritoneum, the transversalis fascia, and the perirenal fascia [14, 15]. The renal fascia divides the retroperitoneal space into three distinct compartments: the posterior pararenal space, the anterior pararenal space, and the perirenal space [15]. 

A review of the literature revealed that the peritoneum and renal fascia play an important role in preventing the spread of disease between the perirenal space and the extraperitoneal pelvis [16]. The anterior and posterior leaves of renal fascia, referred to as Gerota's fascia, merge to form a single multilaminar fascia in the iliac fossa isolating the perirenal space from the extraperitoneal pelvis [16, 17]. However, there is some debate concerning the degree of communication between retroperitoneal and intraperitoneal spaces due to the lack of fusion caudally of the anterior and posterior leaves of Gerota's fascia [16, 18]. The lack of fusion is referred to as an open cone [16]. The open cone concept is supported by studies that show pelvic extension of retroperitoneal fluid may occur by flowing from the infrarenal extraperitoneal space into the pelvic extraperitoneal space [19]. It is likely that the single multilaminated structure formed by the merging of the anterior and posterior leaves of Gerota's fascia contains a potential space that allows some permeability between its layers [15, 16]. Even so, it is rare for perirenal disease to extend into the pelvis and an open cone has not been observed on CT scans [16]. Although there is some communication between retroperitoneal and intraperitoneal spaces due to the lack of fusion of Gerota's fascia, the single multilaminar fascia formed by Gerota's fascia acts as a barrier of disease extension, effectively closing the cone of the renal fasciae [16, 17]. 

As mentioned previously, our patient underwent transabdominal/transperitoneal partial nephrectomy following a stab injury to her left renal artery and left kidney. During this procedure, both the peritoneum and Gerota's fascia were opened, exposing the perirenal space to the extraperitoneal environment and intraperitoneum. A review of the literature reveals that closure of the fascia and peritoneum decrease the risk of tumor seeding and metastasis in experimental models [20, 21]. Furthermore, the literature shows that peritoneal defects may play an important role in tumor implantation and tumor metastasis [22]. It is our understanding that the failure to close the fascia and peritoneum in this patient promoted the tumor expansion from the splenic flexure into the perirenal space and into the kidney.

## 4. Conclusions

Extraluminal colonic carcinomas are a rare tumor entity by itself, and with invasion into the kidney even more. The peritoneum and Gerota's fascia are strong barriers for malignant tumors as seen in daily oncological surgery and *in vivo* experiments. In order to minimize and possibly delay local tumor invasion, the peritoneum and layers of fascia should be closed after surgery in an attempt to restore the normal anatomical situation.

## References

[1] Cappell MS (2008). Pathophysiology, clinical presentation, and management of colon cancer. *Gastroenterology Clinics of North America*.

[2] Brewer DA, Bokey EL, Fung C, Chapuis PH (1993). Heredity, molecular genetics and colorectal cancer: a review. *Australian and New Zealand Journal of Surgery*.

[3] Kibar Y, Deveci S, Sümer F, Seçkin B (2005). Renal papillae metastasis of sigmoid colon adenocarcinoma. *International Journal of Urology*.

[4] Komeya M, Nakaigawa N, Sano F (2009). A case of upper urinary tract metastases from sigmoid colon cancer. *Acta Urologica Japonica*.

[5] Ho L, Wassef H, Henderson R, Seto J (2009). Renal metastasis from primary colon cancer on FDG PET-CT. *Clinical Nuclear Medicine*.

[6] Brambilla E, Heck AA, Cao JG, Toniazzo GT, Petteffi L (2007). Isolated renal metastasis after colon cancer. *The Canadian Journal of Urology*.

[7] Waleczek H, Wente MN, Kozianka J (2005). Complex pattern of colon cancer recurrence including a kidney metastasis: a case report. *World Journal of Gastroenterology*.

[8] Milbank AJ, Savage SJ, Angermeier KW, Ng CS, Streem SB (2004). Metastatic cancer to the renal pelvis: a novel approach to management. *Urology*.

[9] Julianov A, Stoyanov H, Karashmalakov A (2004). Late renal metastasis from sigmoid adenocarcinoma. *Lancet Oncology*.

[10] Aksu G, Fayda M, Sakar B, Kapran Y (2004). Colon cancer with isolated metastasis to the kidney at the time of initial diagnosis. *International Journal of Gastrointestinal Cancer*.

[11] Wolff JM, Boeckmann W, Jakse G (1994). Spontaneous kidney rupture due to a metastatic renal tumour. *Scandinavian Journal of Urology and Nephrology*.

[12] Lowe LH, Zagoria RJ, Chen MYM, Dyer RB (1992). Intraluminal renal metastasis from colon cancer simulating a fungus ball. *Urologic Radiology*.

[13] Shiraishi T, Hasegawa Y, Itoh H, Nakakuki K (1989). Implantation of colon cancer cells onto renal pelvic mucosa. A case report. *APMIS*.

[14] Mirilas P, Skandalakis JE (2009). Surgical anatomy of the retroperitoneal spaces—part I: embryogenesis and anatomy. *American Surgeon*.

[15] Lee SL, Ku YM, Rha SE (2010). Comprehensive reviews of the interfascial plane of the retroperitoneum: normal anatomy and pathologic entities. *Emergency Radiology*.

[16] Raptopoulos V, Lei QF, Touliopoulos P, Vrachliotis TG, Marks SC (1995). Why perirenal disease does not extend into the pelvis: the importance of closure of the cone of the renal fasciae. *American Journal of Roentgenology*.

[17] Chesbrough RM, Burkhard TK, Martinez AJ, Burks DD (1989). Gerota versus Zuckerkandl: the renal fascia revisited. *Radiology*.

[18] Bechtold RE, Dyer RB, Zagoria RJ, Chen MYM (1996). The perirenal space: relationship of pathologic processes to normal retroperitoneal anatomy. *Radiographics*.

[19] Aikawa H, Tanoue S, Okino Y, Tomonari K, Miyake H (1998). Pelvic extension of retroperitoneal fluid: analysis in vivo. *American Journal of Roentgenology*.

[20] Agostini A, Robin F, Jais JP (2002). Peritoneal closure reduces port site metastases: results of an experiment in a rat ovarian cancer model. *Surgical Endoscopy and Other Interventional Techniques*.

[21] Burns JM, Matthews BD, Pollinger HS (2005). Effect of carbon dioxide pneumoperitoneum and wound closure technique on port site tumor implantation in a rat model. *Surgical Endoscopy and Other Interventional Techniques*.

[22] Königsrainer I, Zieker D, Beckert S (2009). Local peritonectomy highly attracts free floating intraperitoneal colorectal tumour cells in a rat model. *Cellular Physiology and Biochemistry*.

